# Characterization of cylindrical ionization chambers for patient specific IMRT QA

**DOI:** 10.1120/jacmp.v10i4.2923

**Published:** 2009-09-30

**Authors:** Danielle Fraser, William Parker, Jan Seuntjens

**Affiliations:** ^1^ Medical Physics Unit McGill University Montréal Québec Canada; ^2^ Department of Medical Physics McGill University Health Centre Montréal Québec Canada

**Keywords:** dosimetry, quality assurance, intensity modulation, ionization chamber

## Abstract

Proven conventional dosimetry techniques do not provide accuracy and precision in the measurement of inverse planned intensity‐modulated radiation therapy (IMRT) fields. Dynamic and step‐and‐shoot multileaf collimation (DMLC/SMLC) challenge current ionization chamber dosimetry practices. Ionization chamber performance in these fields is evaluated for three cylindrical chambers of varying volumes (PinPoint (PP): 0.015 cm^3^, IC10: 0.13 cm^3^, Farmer type NE2571 (FT): 0.69 cm^3^) in terms of measurement reproducibility, dose measurement linearity, and IMRT dose measurements. Fifty IMRT patient specific quality assurance dose measurements were performed with each chamber. DMLC measurements are compared between chambers, and to dose calculations from a commercial treatment planning system (TPS) that used a finite size pencil‐beam model (FSPB). Ten SMLC measurements are compared to Monte Carlo simulations available in the TPS. The three chambers demonstrated adequate measurement reproducibility characteristics for both open and DMLC fields, with each chamber able to perform within 2% (2SD) for DMLC fields. Both smaller volume chambers over responded (>5%) when irradiated with a small number of monitor units in open fields. FT and IC10 chambers demonstrated dose linearity in DMLC fields down to 10 monitor units, while dose linearity for the PP chamber broke down at 100 monitor units. The evaluation of 50 DMLC treatment plan quality assurance procedures revealed that the FT chamber measurements were closest to the FSPB calculated values (FSPB:1.0,FT:0.973±0.044,IC10:0.963±0.048,PP:0.944±0.071). Quality assurance plans calculated independently with Monte Carlo more closely matched chamber measurements (FSPB: 1.0, MC: 0.97, FT: 0.95). Measurements of absorbed dose to water in IMRT fields are highly chamber and IMRT plan dependent.

PACS number: 87.55.Gh; 87.55.km; 87.55.Qr; 87.56.Fc; 87.56.N‐

## I. INTRODUCTION

It is standard practice in radiation therapy to have at least two independent calculations for the number of monitor units (MU) used in patient treatment plans.^(1)^ Measurement‐based and computer‐based checks are two alternative approaches to patient specific quality assurance in intensity‐modulated radiation therapy (IMRT), although the need for treatment plan specificity is debated.^(^ ^2^ ^–^ ^4^ ^)^ The recalculation of one fraction from a patient treatment plan on a phantom, and subsequent measurement, has become the norm for measurement‐based verification, and combines the quality assurance recommendations that deal with the treatment planning system (TPS) and the beam delivery system. This type of evaluation has prompted attention to measurement equipment and techniques. Measurements have been performed with multiple detector types, yet ionization chambers are often still considered the gold standard because of their precision, availability, and relative ease of use.

The use of ionization chambers for the dose verification process in IMRT involves issues particular to the dosimetry of dynamically delivered small fields, varying dose rates, and the summation of multiple low MU segments. Detector characteristics, such as energy dependence, the size of the collecting volume, charge leakage, design, and materials are important considerations.^(^ ^5^ ^,^ ^6^ ^)^ In addition, IMRT dosimetry conditions are radically different from the open field conditions under which the chambers are calibrated, and may invalidate the use of such calibration factors in these radiation beams. Dose discrepancies between the TPS and measurements also arise due to inaccurate beam and component modeling, the dose calculation algorithm, and beam delivery.

This work characterizes three cylindrical ionization chambers of different volumes used for the measurement of dynamic multileaf collimator (DMLC) and step‐and‐shoot (SMLC) IMRT fields. First, an assessment of the reproducibility and dose linearity response of the beam delivery, setup, and measurement systems were made. Second, measurements from all three chambers were evaluated against a commercial TPS pencil‐beam algorithm for 50 DMLC plans. Third, measurements from one chamber were evaluated against the TPS pencil‐beam algorithm and the TPS Monte Carlo (MC) code for ten SMLC plans.

## II. MATERIALS AND METHODS

### A. Measurement equipment

Table 1 lists the properties of the three ionization chambers used in this study: a 0.015 cm^3^ PTW‐Freiburg 31006 PinPoint (PP) (PTW‐Freiburg, Freiburg, Germany), a 0.13 cm^3^ Wellhöfer IC10 (Scanditronix Wellhöfer North America, Bartlett, TN), and a 0.69 cm^3^ Farmer‐type (FT) ionization chamber model NE2571 (Nuclear Enterprises, Fairfield, NJ). Rectangular slabs of Solid Water (Gammex‐RMI, Middletop, WI), RMI 457 certified grade, were used as solid phantom materials. The phantom was constructed of three slabs of solid water and had total dimensions of $30\times 30\times 17\;{\text{cm}}^{3}$. A hole was milled in the center slab to accept a custom shaped close fitting Solid Water sleeve constructed for each ionization chamber. The chamber's sensitive volume was thus located at the center of the phantom. The three phantom and chamber combinations underwent a computed tomography (CT) scan, which was used by the TPS to calculate the dose to the chamber.

**Table 1:** Cylindrical ionization chamber construction properties.

*Ionization Chamber*	*PinPoint*	*IC10*	*Farmer‐type (NE2571)*
Nominal sensitive volume	0.015 cm^3^	0.13 cm^3^	0.69 cm^3^
Sensitive volume length	5 mm	5.8 mm	24.1 mm
Inner diameter of outer electrode	2 mm	6 mms	6.3 mm
Wall material	PMMA $1.19\;g/{\text{cm}}^{3}$, graphite $0.82\;g/{\text{cm}}^{3}$	Shonka C552 $1.76\;g/{\text{cm}}^{3}$	graphite $0.82\;g/{\text{cm}}^{3}$
Wall thickness	PMMA 0.56 mm graphite 0.15 mm	0.4 mm	0.36 mm
Inner electrode material	Steel	Shonka C552	aluminum
Length of inner electrode	4.5 mm	3.3 mm	20.5 mm

All measurements were performed with a 6 MV photon beam on a Varian CL21EX linear accelerator fitted with a Varian Millennium 120 leaf collimator (Varian Oncology Systems, Palo Alto, CA). The output of the linac was previously calibrated using the TG‐51 protocol under TG‐51 reference conditions.^(7)^ The nominal dose rate for all measurements was set at 400 MU/min. However, the actual DMLC dose rate dropped to an average of 200 MU/min due to a restriction on the maximum leaf speed.

The chambers were cross‐calibrated with a secondary standard at our clinic for the photon beam in question. The secondary standard is the clinic chamber that was sent to a primary standards lab for calibration. Under standard open field conditions, the absorbed dose‐to‐water calibration coefficient, $N_{\text{Dw}}$, was determined. The dose‐to‐water from ionization chamber measurements was calculated by multiplying the corrected raw ionization signal (corrected for charge leakage, environmental conditions, and daily linac output variation) with $N_{\text{Dw}}$.

### B. Treatment planning system

Treatment plans of a cohort of 50 patients (head and neck, and pelvis) were randomly selected for retrospective verification in this study. The CORVUS inverse treatment planning system, version 5.0R5 (NOMOS Corporation, Cranberry, PA) was used for plan optimization and finite size pencil‐beam (FSPB) dose calculations.^(8)^ The beamlet cross section used for the calculation was $10\times 5\;{\text{mm}}^{2}$. PEREGRINE, version 4.0 (NOMOS Corporation, Cranberry, PA), was used for MC dose calculations.^(9)^ PEREGRINE is a subsystem of CORVUS offering an alternative MC‐based dose recalculation tool. PEREGRINE is based on the EGS4/BEAM^(10)^ codes for source matching, and employs the 1.6E2 MLC component module previously optimized to match experimental leakage profiles. Details on the PEREGRINE code and simulations are provided in Heath et al. (2004).^(11)^ The uncertainty of PEREGRINE open field simulations was chosen to be 0.1% of the maximum dose and required 3 hours of computation time on a cluster of 8 dual 800 MHz computer processors. Patient dose calculations were calculated to 0.5% uncertainty and required approximately 12 hours of computation time. The final PEREGRINE and CORVUS dose matrices were interpolated to the resolution of the CT matrix, 0.742 mm/pixel. However, the TPS only displays a dose resolution of 1 cGy.

The CORVUS and PEREGRINE algorithms have previously been verified against standard data provided in protocols, and against measured data at multiple institutions.^(^ ^11^ ^–^ ^16^ ^)^ Heath et al. (2004) stated an average agreement of 3% between PEREGRINE and measurements in static and dynamic MLC defined fields, and Boudreau et al. (2005) stated an average agreement of approximately 2% between PEREGRINE and CORVUS (using the electron path length heterogeneity correction) for the mean dose to the CTV for head and neck cases.

### C. Hybrid plan creation

To assess each chamber's performance in DMLC quality assurance procedures, one fraction from each of 50 patient treatment plans was delivered once to each ionization chamber and compared to TPS calculated doses. The TPS calculated dose to the chamber is called a hybrid phantom plan. Hybrid plans use the original patient beam geometries (MLC sequencing, gantry angles, and monitor units) to calculate the dose on CT images of the chamber in the phantom. For ionization chamber quality assurance, the volume of interest is the chamber sensitive volume as contoured on CT images of the chamber in the Solid Water phantom. Calculated dose statistics were averaged over each chamber's delineated pixel volume; thus the volume of interest, and calculated dose, were different for each chamber. Three hybrid phantom plans (one for each ionization chamber) were calculated for each patient plan. For FSPB dose calculations, heterogeneities were not taken into account in order to mimic the protocol used in the clinic at our institution. Hence the phantom and chamber material were assumed to be a water‐equivalent homogeneous slab. The ionization chamber volume was always located in a region of high dose and low dose gradient (less than ±5%).

Hybrid plan dose calculations with PEREGRINE did consider heterogeneities and each CT voxel was assigned both a medium type and an electron density value (relative to water) based on a calibration curve derived from CT Hounsfield units. It was noted that the contoured chamber volumes contained primarily lung‐type, muscle‐type, and bone‐type tissues, and only a few of the pixels within the contoured volumes were labeled as air. The resolution of the calculation dose matrix and CT pixel sizes may not have been able to resolve material type in the small chamber volume. For these reasons, comparisons with MC were normalized to open field conditions.

#### D.1 Measurements of reproducibility and linearity

For each of the three cylindrical ionization chambers used in this study, a reproducibility experiment was performed for a $10\times 10\;{\text{cm}}^{2}$ jaw‐defined open field, and the same randomly chosen DMLC hybrid plan. In the open field, 200 MU were delivered at a dose rate of 400 MU/min while, for the hybrid plan, the MUs were the same as those prescribed in the patient treatment plan. Hybrid plan measurements were taken by aligning each chamber in the phantom on the treatment couch at the selected point of measurement in the plan distribution. Intersession reproducibility, in which a new phantom setup was performed, was evaluated and provided a measure of the consistency of machine output, MLC leaf positioning accuracy, stability of detector response, and phantom positioning. Each intersession measurement is an average of ten intrasession measurements during which the phantom setup was undisturbed and only the beam was redelivered. We define the uncertainty of measurements as two standard deviations (SD) in order to contain 95.5% of all measurements.

A dose linearity assessment was performed using an open field and the same randomly chosen DMLC field. MU settings between 1 and 500 MU were used, and the resulting charge readings were corrected and normalized in terms of nC/MU.

#### D.2 Measurements of DMLC delivery

Fifty DMLC hybrid plans were delivered once to each ionization chamber and compared to the FSPB calculated dose. The beams were delivered to the phantom exactly as they were to the patient.

#### D.3 Measurements of SMLC delivery

Ten SMLC plans were selected for comparison with PEREGRINE and delivered once to the FT chamber. For technical reasons the MC algorithm was unable to model DMLC delivery, so SMLC sequences were used. This required reoptimization of the MLC sequences. Comparisons of measurement against FSPB and PEREGRINE were performed.

## III. RESULTS & DISCUSSION

### A. Reproducibility

**Figure 1:**
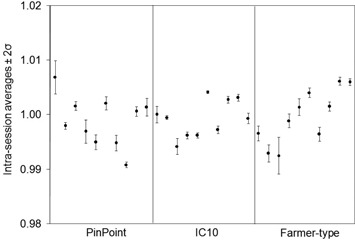
Reproducibility of ionization signals for a $10\times 10\;{\text{cm}}^{2}$ open field. Each point is the intrasession average, normalized to the intersession average. The error bars are ±2SD of the intrasession measurements, normalized to the respective intrasession average.

**Figure 2:**
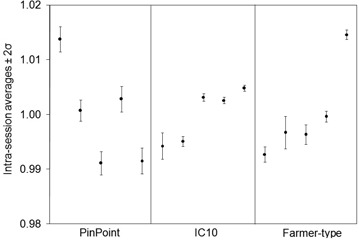
Reproducibility of ionization signals for one dynamic multileaf collimator hybrid plan fraction. Each point is the intrasession average, normalized to the intersession average. The error bars are ±2SD of the intrasession measurements, normalized to the respective intrasession average.

The results for the reproducibility experiments are shown in Table 2 and Figs. 1 and 2. Each data point is the intrasession average, normalized to the intersession average. The error bars are ±2 standard deviation of the intrasession measurements, normalized to the respective intrasession average.

**Table 2:** Intersession reproducibility (2SD) for a $0\times 0\;{\text{cm}}^{2}$ open field and a DMLC field.

		*PinPoint*	*IC10*	*Farmer‐type*
$0\times 0\;{\text{cm}}^{2}$ field	intersession 2SD (%)	1.2	0.7	1.0
DMLC field	intersession 2SD (%)	1.9	1.0	1.7

The percentage uncertainty on intrasession measurements (indicated by the error bars in Figs. 1 and 2) ranged up to 0.2% for the open field and up to 1.0% for the DMLC field. The difference in the delivery between these two fields is the movement of MLC leaves, indicating that MLC positioning is not as consistent as machine output and detector response combined. The percentage uncertainty on intersession measurements for the open and DMLC hybrid plan for the PP, IC10, and FT chambers were 1.2% and 1.9%, 0.7% and 1.0%, and 1.0% and 1.7%, respectively. The intersession standard deviation values are larger confirming that chamber positioning plays a substantial role in measurement uncertainty. The smallest chamber is more sensitive to geometrical positioning within the treatment field, while larger chambers will average the dose over their sensitive volume and be even less representative of the dose at a point. On average, the IC10 experienced the least amount of intersession fluctuation among the three chambers.

### B. Linearity

**Figure 3:**
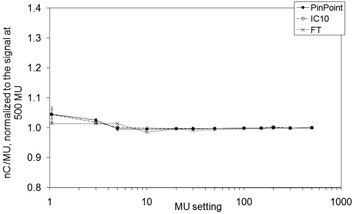
Dose linearity for a $10\times 10\;{\text{cm}}^{2}$ open field at a dose rate of 400 MU/min. The ionization signals ±2SD per MU, normalized to the signal at 500 MU, are plotted.

**Figure 4:**
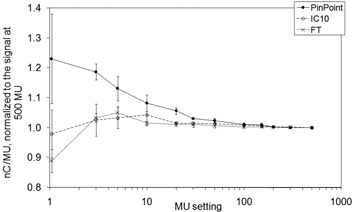
Dose linearity for one dynamic mulitleaf collimator hybrid field at a nominal dose rate of 400 MU/min. The ionization signals ±2SD per MU, normalized to the signal at 500 MU, are plotted.

The results for the dose linearity measurements are shown in Figs. 3 and 4. The corrected charge readings per MU, normalized to the values at 500 MU, are graphed. In fields with dynamically moving leaves, the MU setting not only dictated the dose to be delivered, but also the leaf speed and dose rate. In order to meet the control points during IMRT delivery, leaf speed and dose rate are changed on the fly, and the reproducibility of control point matching is limited by the leaf positioning tolerance level. At low MU settings in the DMLC field, the smallest volume chamber's response increased by 23%, while the largest volume chamber's response decreased by 11%. For low MU settings in accelerator IMRT mode, the dose rate is automatically adjusted by the accelerator workstation when the maximum leaf speed is reached so that the leaves meet their next control points. For greater MU settings, the leaf speed is reduced while maintaining the nominal dose rate of 400 MU/min. At 3 MU in the open reference field, the signal per MU changes for all three chambers, compared to the corresponding changes in signal per MU in the hybrid field at 150 MU. Starting immediately at 150 MU in the hybrid field, the dose rate dropped from the nominal setting. As the dose, and subsequently the dose rate, was reduced further, the proportional amount of time to complete delivery increased, so that leakage corrections, based on the observed current with the beam off, became a relatively larger portion of the total signal. At 500 MU, the leakage contribution for the PP chamber in the open and DMLC field was 0.3% and 0.6%, respectively. At 1 MU, the leakage contribution in the open and DMLC field was 0.3% and 16%. In similar IMRT field studies, the incorporation of charge leakage in measurements from an Exradin A1 0.009 cm^3^ micro chamber altered calculated IMRT doses by up to 16%.^(17)^ On the other hand, the contribution to the IC10 signal at 1 MU was 0.3% for the open field compared to 2% for the DMLC field. Although treating a patient by delivering 1 MU in a DMLC field is not practical, the use of highly conformal techniques increases the number of beams such that the number of MUs per beam decreases.

Effects contributing to non‐linear output may be highly dependent upon the fluctuation of instantaneous dose rate with respect to MLC leaf position over the chamber volume, which is altered on the fy. For example, the extreme case of delivering 1 MU demonstrates that forced low dose rates may cause radiation producing active pulses to be delivered irregularly during an IMRT field, and may not coincide with the location of the chamber. However, their impact on the total signal for a full IMRT delivery may be small, as measurements are integrated over multiple fields.

### C. Evaluation of ionization chambers for DMLC QA measurements

**Figure 5:**
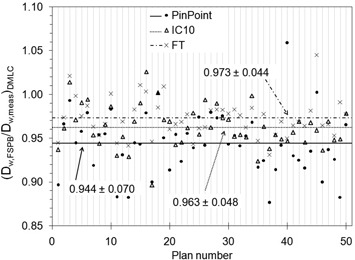
Ratios of absorbed dose‐to‐water from the finite size pencil‐beam (FSPB) algorithm to measurement for 50 dynamic mulitleaf collimator (DMLC) hybrid plan fractions. The averages are marked with horizontal lines.

Figure 5 compares the ratio of absorbed dose‐to‐water from the FSPB calculation to that from measurements, ${\left(D_{w,\text{FSPB}}/D_{w,\text{meas}}\right)}_{\text{DMLC}}$, for 50 DMLC plans. The mean ratios and uncertainties are 0.944±0.070 for the PP, 0.963±0.048 for the IC10, and 0.973±0.044 for the FT. For these dynamic hybrid plans, the FSPB systematically underestimated the dose compared to measurements. The degree of underestimation was the greatest for the smallest volume chamber. Factors that contribute to the comparison process can be divided into two categories: those involved in the CORVUS FSPB calculation, and those involved in the measurement.

The accuracy in the TPS prediction is a function of how well the analytical model reproduces various accelerator beam geometries and how well it reports dose‐to‐water in IMRT fields. Inconsistencies were already observed under reference conditions of a $10\times 10\;{\text{cm}}^{2}$ field where the FSPB overestimated the absolute dose by about 2% for the three ionization chambers. The dose statistics calculated by the TPS over the chamber volume are dependent upon the digitally contoured volume, which was delineated from visual inspection of a CT scan of the phantom and chamber (and was larger than the manufacturer's nominal chamber volume due to slice averaging effects present in CT images). Smaller beamlet cross sections may be able to calculate the dose more accurately.^(18)^ For instance, the beamlet size used in this work had an area greater than the cross section of the smallest volume chamber. In addition there are approximations specifically related to the effects of the MLC such as penumbra, rounded leaf ends, intra and interleaf leakage, and the tongue and groove design. The impact of the MLC on dose distribution features and the applicability of broad beam data for commissioning may be more complex in IMRT fields, and indicate an inadequacy with the pencil‐beam algorithm. Even if the TPS algorithm accurately calculates the dose inside the cavity volume, the transfer of planned beam fluences to delivery may not be accurate itself. This study assumed that the TPS converted the optimized fluence intensity map into an MLC sequence exactly.

We must also consider uncertainties in the measurement process when assessing ionization chamber performance. The PP chamber experienced the largest variation in measured dose both in terms of absolute deviation (as seen in Fig. 5) and in terms of standard deviation (as seen in Table 3). The PP systematically measured larger doses than the other two, even though it has been suggested that small volume chambers may not detect contributions from remote IMRT fields due to low sensitivity.^(5)^ The discrepancy in PP measurement may be due to detector design and construction materials, as some have reported that the PP overresponds to low‐energy Compton scattered photons due to a steel central electrode.^(6)^ When compared to conventional fields, fields with moving leaves contain a larger proportion of scattered photons to primary photons because of less sharp penumbras due to rounded leaf edges that travel across the entire field. The deviations may also be due to a high sensitivity to positioning accuracy, and the fact that the cross section of the TPS beamlet was larger than the PP's volume. However, because the chambers have been cross‐calibrated in open field geometry, the variations in measured doses and large standard deviations of Table 3 may point to chamber specific responses in IMRT dose delivery for the same beam configuration.

**Table 3:** Evaluation of ionization chambers for DMLC QA measurements.

	*Average (range) (cGy)*		Average±2SD(cGy)
*Chamber*	*FSPB Algorithm*	*Measurement*	${\left(D_{w,\text{FSPB}}/D_{w,\text{meas}}\right)}_{\text{DMLC}}$
PinPoint	200.9 (101.7, 277.1)	211.5 (109.8, 302.5)	0.944±0.070
IC10	200.5 (103.1, 277.1)	208.5 (108.7, 293.1)	0.963±0.048
Farmer‐type	200.1 (105.0, 276.9)	207.3 (110.1, 287.2)	0.973±0.044

The average dose calculation and measurement are presented in columns two and three. The average dose ratios ±2SD are presented in column four.

Another characteristic of the data in Fig. 5 is that there are some chamber measurements that differ substantially from other chamber measurements – outliers. It is clear that the PP's outliers are greatest in number and magnitude. From the reference field and dynamic hybrid field tests, it can be ruled out that intersession fluctuations affected measurements to such a degree as to account for the large discrepancies observed over this population of 50 IMRT plans. Again, these outliers may be due to a high sensitivity to position within the treatment field, the inability of the TPS to calculate the dose to such a small volume, or detector construction and materials.

During dynamic MLC IMRT plan delivery on a rectangular phantom, three dosimetric parameters of interest changed: dose rate, field size, and depth of measurement. The field size and depth of measurement will alter the scatter particle fluence reaching the detector. During IMRT delivery, the detector is often located either outside of the field or in penumbra regions, resulting in volume averaging (especially important over gradient regions). Partial volume irradiation leads to lateral electronic disequilibrium where the nonuniform response to exposure within the detector's volume spatially affects the conversion of signal into dose‐to‐water in the detector.

In absorbed dose‐to‐water based protocols, the absorbed dose‐to‐water calibration coefficient incorporates fluence perturbation correction factors. However, IMRT fields produce beam fluences that differ substantially from calibration conditions.^(19)^ For example, $P_{\text{fl}}$ and $P_{\text{gr}}$ are affected by the degree of lateral electronic disequilibrium at the field edges. The measured dose is also representative of the dose at a point upstream, along the incident particle's direction, from the physical location of the center of the collecting volume in a homogeneous phantom. If irregular dose gradients are present over the chamber volume as a result of complex modulation, the exact location of the effective point of measurement is uncertain. The application of static reference field conversion and correction factors to IMRT fields may introduce considerable errors in dose determination. In fact, as has been noted, determining these factors is heavily dependent upon the geometry and beam characteristics specific to each IMRT field.^(^ ^20^ ^,^ ^21^ ^)^ Given that the chamber‐specific dose ratios with the FSPB algorithm are farther from unity than the chamber‐to‐chamber comparisons, the beam delivery and measurement processes do not fully account for the discrepancies in Fig. 5.

**Figure 6:**
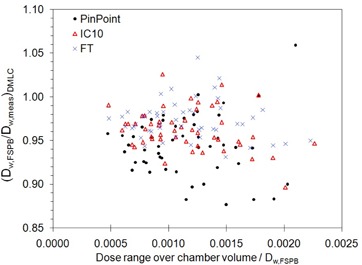
Ratios of absorbed dose‐to‐water from the finite size pencil‐beam (FSPB) algorithm to measurement, as a function of the FSPB predicted dose range over the chamber volume in dynamic mulitleaf collimator (DMLC) hybrid plan fields. The range has been normalized to the FSPB predicted dose.

In addition to predicting the average dose over the chamber volume, the TPS also calculates the range of doses (minimum to maximum) and standard deviation over the same volume. Figure 6 compares the ratios of FSPB dose calculation to measurement, as a function of the maximum dose range (normalized to the average calculated dose) over the chamber volume – the latter parameter quantifying the relative dose gradient over the chamber. It can be seen that the ratios deviate further from their respective averages as the dose gradient increases. In this context, a new formalism for calibration of dynamic fields where reference fields are defined that minimize gradients over the measurement device has been presented by Alfonso et al.^(22)^ and is expected to significantly improve dosimetric accuracy.

In practice, absolute dose discrepancies between a treatment planning system and measurement can be clinically accounted for with a single TPS “fudge” (correction) factor that adjusts the analytical algorithm to better match systematically different IMRT measurements.^(23)^ However, Fig. 5 shows that the factor is chamber‐dependent, and the large two standard deviations (7.0 % for the PP, 4.8% for the IC10, and 4.4% for the FT) indicate that the FSPB algorithm cannot be altered by a single factor to accurately match all (95.5%) measurements. Alternatively, treatment plan specific correction factors can be applied to each ionization chamber to account for fluence perturbation effects in individual segments of an IMRT delivery.^(20)^

### D. Evaluation of SMLC QA measurements

**Figure 7:**
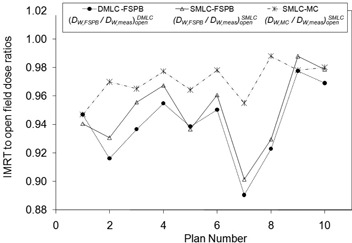
Ratios of FT measurements to TPS dose, for DMLC and SMLC modes, normalized to open field conditions.

The doses measured from ten quality assurance plans delivered with SMLC beams were compared with both the FSPB algorithm and MC techniques (Fig. 7 and Table 4). Ideal matching between TPS and measurement would result in dose ratios of unity, but both FSPB and MC underestimated the measured dose in IMRT fields. FSPB calculations and measurements agreed slightly better for SMLC delivery than for DMLC delivery. MC calculations showed superior matching with measurements by more than 2% on average compared to FSPB calculations in SMLC delivery and, with only a 2.5% spread in the data, the MC code may also have been able to account for treatment plan specific characteristics.

**Table 4:** Summary of results for ten DMLC hybrid plans (measurement to FSPB) and ten SMLC hybrid plans (measurement to FSPB and measurement to MC).

*10 Dynamic*	*10 Step‐and‐Shoot*	
${\left(D_{W,\text{FSP}B}/D_{W,FT}\right)}_{\text{ope}n}^{\text{DML}C}$	${\left(D_{W,\text{FSP}B}/D_{W,FT}\right)}_{\text{ope}n}^{\text{DML}C}$	${\left(D_{W,MC}/D_{W,FT}\right)}_{\text{ope}n}^{\text{DML}C}$
0.945±0.057	0.949±0.053	0.970±0.025

Comparisons are made with the TPS calculation and measurement ratios in IMRT fields to $0\times 0\;{\text{cm}}^{2}$ open fields. The average ratios over all plans ±2SD are presented.

## IV. CONCLUSIONS

Fifty dose measurements, each with three different ionization chambers, were compared to a FSPB algorithm and a commercial MC engine. It was demonstrated that different chambers respond individually to DMLC field conditions where the larger volume chamber consistently measured lower doses than the smaller volume chambers. All chambers measured higher doses than those predicted by both FSPB and MC. However, MC calculations better matched measurements than did FSPB calculations in SMLC delivery by more than 2% on average, and the standard deviation of MC to measurement was approximately half that of the FSPB. These average dose discrepancies are all greater than that estimated from the reproducibility study and greater than the uncertainty used in MC dose calculations. The implementation of a TPS calibration factor would center the average discrepancy between measurement and TPS at zero and remain within ICRU Report 24^(24)^ recommendations for clinical accuracy of ±5%, assumed to be at the 1.5–2 standard deviation level. More importantly, due to the uncertainty in TPS calculations, the chamber specific measured dose dependency, and the uncertainty in the conversion of ionization signal to absorbed dose‐to‐water in IMRT fields, the true dose to the chamber is not known with certitude.

## ACKNOWLEDGEMENTS

This work was partially supported by CIHR grant MOP 57 828. Jan Seuntjens is a research scientist at the National Cancer Institute of Canada, supported with funds of the Canadian Cancer Society.
